# Seasonal body image dissatisfaction: a bi-hemispheric panel analysis of social media users across 4 years

**DOI:** 10.1007/s40519-025-01782-9

**Published:** 2025-08-31

**Authors:** Justin Thomas, Timothy Regan, Rana Samad, Yasmin Aljedawi, Dahlia AlJuboori, Alex Wells

**Affiliations:** 1 Global Digital Wellbeing Program (Sync), King Abdulaziz Center for World Culture (Ithra), Dhahran, Saudi Arabia; 2 Department of Medical and Clinical Psychology, School of Medicine, Uniformed Services University of the Health Science, Washington, D.C., USA; 3 Department of Mental Health, Johns Hopkins Bloomberg School of Public Health, Washington, D.C., USA; 4 Depertment of Primary Care and Mental Health, University of Liverpool, Liverpool, UK

**Keywords:** Social media, Body image, Big data, Eating disorder, Seasonality

## Abstract

**Purpose:**

Seasonal body image refers to within-person variations in body image satisfaction that correspond with climatic seasonality (winter, spring, summer, and autumn). Previous cross-sectional research involving male participants from northern (UK, USA, and Canada) and southern hemisphere (Australia) nations reports a peak in body image dissatisfaction during the summertime, with a decrease in the wintertime. Big Data extracted from social media platforms provides a novel means of further exploring the seasonal body image hypothesis in a larger and more diverse sample across several years.

**Methods:**

This study utilised panel data drawn from X/Twitter, a social media platform, to investigate the posts (*N* = 12,017,766) of users/authors (*N* = 1534) between 2020 and 2023. The panel consisted of authors from countries in both the northern and southern hemispheres. A template-driven search algorithm identified expressions of body image dissatisfaction (BID) in users’ posts.

**Results:**

The rate of BID (relative to the overall number of posts) was calculated for each calendar month. A statistically significant summer spike was observed in the Northern hemisphere, while the data were non-significant but directionally supportive of a similar summer spike in the Southern hemisphere.

**Conclusions:**

This study partially supports the seasonal body image hypothesis, adding nuance to the current understanding of seasonality. This research has implications for the timing of public health initiatives aimed at preventing body image issues and eating disorders.

**Level of evidence IV:**

Evidence obtained from multiple time series without intervention.

**Supplementary Information:**

The online version contains supplementary material available at 10.1007/s40519-025-01782-9.

## Introduction

### Body image dissatisfaction

The latter half of the twentieth century witnessed a proliferation and globalisation of body image dissatisfaction [1]. Gordon [2] describes this phenomenon as the rise of the thin body cult, pointing to societies, where extremely thin and tubular (low waist-to-hip ratio) female body images have become idealised amidst rising societal obesity. Illustrating a similar point, Garner et al. [3] report decreasing weight and body dimensions of Miss America beauty pageant contestants between 1959 and 1978, co-occurring within the context of increasing weight norms for US women. A more recent study examined Victoria’s Secret models between 1995 and 2018, reporting the same phenomenon: shrinking ideals (models) alongside expanding norms [4]. Such discrepancies between body image aspirations/ideals and anthropometric realities (or subjective estimations) are central to most definitions of, and attempts to measure, body image dissatisfaction [5–9].

### Prevalence and potential implications

Prevalence studies of body image dissatisfaction routinely report rates exceeding 50%, particularly among college-age women [10–13]. Such high rates have led to the notion of normative discontent, where body image dissatisfaction is the rule rather than the exception [5]. Although more frequently reported in women [14], the disparity between body image ideals and actual (or perceived) body weight and shape is also increasingly reported among men [15, 16].

Body image dissatisfaction and extreme body image attitudes, for instance, overvaluing weight and shape [17], are associated with maladaptive weight-control measures [18] and eating disorders, such as anorexia and bulimia nervosa [19]. Numerous studies report an elevated prevalence of eating disorder symptomatology among subpopulations and occupations, where extreme thinness (the attainment of body image ideals) is a prerequisite for inclusion or success. For instance, Preti et al. [20] reported elevated rates of eating disorder psychopathology among Italian fashion models compared to age-matched controls. Other earlier studies report similar findings for aspiring ballerinas and athletes engaged in sports that emphasise thinness to facilitate performance or aesthetics, such as gymnastics or figure skating [21]. In short, extensive clinical experience [22], along with primary [23] and secondary research [24, 25] suggests body image dissatisfaction is a risk factor for the onset and maintenance of eating pathology.

### Seasonal body image

Body image dissatisfaction can be conceptualised as an enduring trait that is relatively stable across time [26], referring to one’s typical feelings about their body. However, it can also be viewed and assessed as a state-like phenomenon, fluctuating throughout the day and across various contexts [27]. For instance, one study employed an ecological momentary assessment methodology over seven days and found that levels of body image satisfaction varied across time, with increased dissatisfaction linked with negative social interactions and predictive of avoiding subsequent social interactions. [27]. Furthermore, exposure to societal body image ideals (e.g., thin models) via traditional [28] or social [29] media appears to increase feelings of body image dissatisfaction among women and men [30]. One proposed mechanism for media-induced body image dissatisfaction is rooted in social comparison theory [31]. Experimental studies have demonstrated that upward social (body image) comparison—comparing one’s body shape and size to that of another person deemed to be in better shape—results in lower body satisfaction. Interestingly, downward comparisons did not result in uplifts in body image satisfaction, but were associated with increased anxiety [32]. Social media platforms present users with the opportunity to share and view other people’s visual content (videos and photographs) affording ample opportunity for body image-related social comparisons, with the potential to negatively impact body image satisfaction.

In addition to the impact of upward social comparison on body satisfaction, Griffiths et al. [33] proposed that body image dissatisfaction might also fluctuate according to a seasonal pattern, intensifying in the summer and attenuating during the winter. They found some support for this idea of seasonal variation in levels of body image dissatisfaction in a cross-sectional study of sexual-minority men residing in the USA, UK, Canada and Australia. The authors propose that the mechanisms driving the summer spike in BID include climate-related social activities, such as beach-going and swimming. Along with the hotter temperature, these pastimes generally require people to wear less and more revealing clothing. In addition, the authors argue that these climatic changes (less rain, more sun) also give rise to appearance-focused media advertising (e.g. “Are you beach body ready?”), with similar peer-related pressures reflected on social media, such as summer shredding challenges, where shredding equates to losing fat while maintaining muscle [33].

Very few studies (we can identify only one) have explored seasonal body image dissatisfaction (within-person variation in body image dissatisfaction across four climatic seasons). Such research has the potential to inform the timing of eating disorder prevention campaigns and other associated public health initiatives. Griffiths et al.’s study was limited to sexual-minority men across a single calendar year. Social media data sets (big data) offer a promising and complementary window for exploring the idea of seasonal body image further, addressing some of the limitations of earlier work in this area.

### Big data in public health research

The National Science Foundation (NSF) describe big data as “large, diverse, complex, longitudinal, and/or distributed data sets generated from instruments, sensors, Internet transactions, email, video, click streams, and/or all other digital sources available today and in the future”[34]. Big data are also frequently characterised using the three vs. acronym: volume, velocity and variety. Specifically, these are large, computerised data sets (volume) that grow rapidly in size and complexity over time (velocity), accommodating multiple data types (variety), such as images, text, and audio [35].

The potential value of big data as a complement to traditional research methods in cognitive, social, and health sciences has been widely appreciated [36–38]. Oswald [39] notes that, as a professional group, psychologists have extensive training in extracting substantive understanding from findings based on complex data modelling involving many variables, some of which are hard to measure. In this regard, big data extracted from social media platforms has been of particular interest as both communication content (i.e., text, images, and videos) and social networks (i.e., individual users within group memberships, interactions between in-group and out-group members) can reveal insights about complex behaviour and interpersonal processes with implications for public health issues [40].

The application of big data analytics to social media data for this purpose has proven fruitful. Thorstad & Wolff [41], for instance, combined textual analysis of Reddit posts with machine learning clustering and classification analyses to gain insight into language signals that predict future mental health issues. They discovered that language associated with life stress posted to non-clinical subreddits (e.g., cooking, cars, and travel) was predictive of users’ future posting to clinical subreddits (e.g., ADHD, anxiety, and bipolar). Similarly, Valdez et al. [42] applied machine learning algorithms to analyse Twitter data during the initial stages of the COVID-19 pandemic. They found differences in the emotional content of March 2020 tweets, such as the sentiment of COVID-19-related tweets overall trended toward positivity and optimism over the month, but individual user timelines of all tweet content (not just COVID-19-related) indicated the sentiments within individuals trended toward negativity and pessimism, uncovering inconsistencies between public portrayals of well-being vs. potential underlying mental distress. Similar studies have used social media data to detect shifts and signs of potential widespread mental health issues, such as depression, suicidal ideation, and disordered eating [43–45].

### Research question

This study used social media data from Twitter (renamed X in July 2023) to explore the seasonal body image hypothesis. Using data from both northern and southern hemisphere nations, we were able, according to respective meteorological conventions, to formulate hemisphere-specific hypotheses regarding which months might be linked to an increase in expressions of BID. Measuring the same individuals over multiple calendar years extends the work of Griffiths et al., enhancing the accuracy of seasonal body image assessment by capturing monthly within-person variation in spontaneous expressions of body image dissatisfaction. The study’s two primary hypotheses are (H1) that the northern hemisphere’s summer months—June, July, and August combined—will exhibit a higher rate of BID as expressed through social media posts than the winter months (December, January, and February). Similarly, (H2) we hypothesise that the southern hemisphere’s summer months—December, January, and February—will also show a higher rate of BID compared to the winter months (June, July, and August).

## Methods

### Data acquisition

Data collection from X (formerly Twitter) was facilitated directly through X’s Enterprise Application Programming Interfaces (APIs), software applications allowing access to real-time and historical data from 2006 onward. The present study used data obtained via Pulsar, a social listening and advanced audience analytics company. The data were extracted using the “Firehose” API, which combines the filtered stream API for live tweets and the Search API for past data. The data made available via the Firehose API includes posts, reposts, quotes, replies, and engagement metrics, such as likes and impressions. Metadata such as location, language, and user demographics are also provided. Data from blacklisted accounts (including suspended or deleted accounts) or containing excluded search terms is not provided. It is also important to note that demographics such as age and sex are not explicitly recorded in Twitter accounts. However, sex/gender can typically be inferred from the biographical text that users supply.

### Panel selection (participants/authors)

Based on available data processing capacity, we aimed to identify 2,000 active Twitter users (authors) who had expressed body image dissatisfaction (BID) at least once during the 4-year study period (from January 1, 2020, to December 31, 2023). Expressions of BID were detected using an English language-based, template-driven search strategy. This algorithm involved using templates, such as “hate the way my ______ looks” and “not happy with my_____”, which were iteratively completed with a list of appearance-related stems, such as “body,” “thighs,” “hips,” etc. For full details of the templates and stems used, see online Appendix 1. Examples of tweets that were screened as expressions of BID include the following (spelling corrected and slightly modified to preserve anonymity): “I have not worn leggings in over a year because of how much I hate my legs”, “.. my body image is becoming such a massive issue at the moment it’s on my mind constantly..”, “My body image was horrendous, now it’s even worse” and “ I hate my legs. My upper body is like average, but my legs look like they belong on an overweight teen”. We included authors from five nations, where English is widely spoken: two Northern Hemisphere nations (the UK and the USA) and three Southern Hemisphere nations (Australia, New Zealand, and South Africa). From the initial data set of matching posts, authors with unusually large follower counts (exceeding one standard deviation from the mean) were removed to quickly exclude celebrities and influencers who frequently use social media platforms as part of their personal brand strategy.

Finally, a manual tagging process was conducted to identify individual accounts (users) eligible for panel inclusion. There were 60,014 candidate users for the Northern Hemisphere and 1488 from the South (see Table 1). These candidate users were randomly assigned and then presented to a human analyst (the fourth author of this paper at the time, who was blind to the study hypotheses). The data included username, user biography, follower count, and all relevant posts matching the search criteria. The analyst evaluated each user and their posts, with instructions to exclude organisations, commercial entities, and individuals discussing the topic from professional, medical, or academic perspectives. The analyst was instructed to continue tagging until either 1,000 eligible users were identified or no further candidates remained in the data set. This process resulted in 556 users from the Southern Hemisphere and 1000 from the North (see Table 1). This manual tagging process also involved assigning male/female sex based on the user’s name or sex mentioned in the user’s biography. Age and sex are not included as fields in the X/Twitter data set.

**Table 1:** Panel acquisition steps with associated frequency counts

Panel acquisition steps	Northern panel	Southern panel
Number of users contributing at least one post matching the template search (expressing BID)	60,014	1488
Number of posts matching template search	66,571	1645
Number of manually verified users included	1000	556
Percentage female	53.69	47.6
Total posts published by selection of relevant users	6,367,148	5,650,518
Posts expressing BID	690	620

The final data set included a monthly count of all posts per user alongside a monthly count of each user’s BID posts. Retweeted posts were removed (retweeting = reposting one’s own or another user’s posts).

## Results

All data were positively skewed; consequently, we employed non-parametric tests when making inferences. Expressions of BID occurred at 0.1083 per 1000 tweets for the northern hemisphere panel, while a slightly higher rate of 0.1097 per 1000 was recorded for the southern hemisphere panel. We then compared the rate of BID tweets by season, calculating Northern Hemisphere summer as the sum of BID tweets for June, July, and August, and winter as the sum for December, January, and February. For the Southern Hemisphere, this pattern was reversed.

### Northern hemisphere

**Fig. 1:**
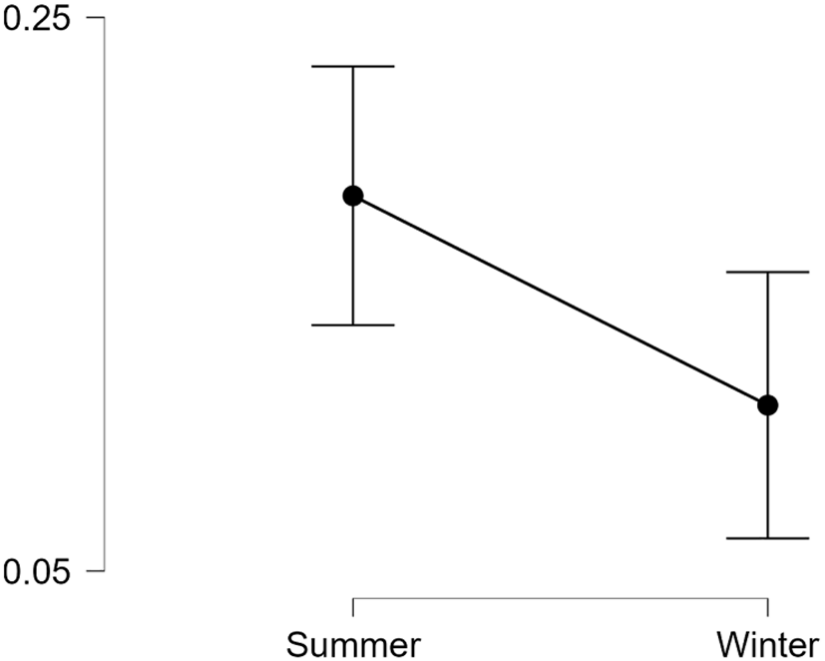
*Mean BID Tweets per 1000 by Season for the Northern Hemisphere Panel.* Summer months are Jun, Jul and Aug, with Dec, Jan, and Feb considered winter months

In the Northern Hemisphere, the rate of BID tweets was higher during summer (MDN = 0.00221) compared to winter (MDN = 0.00210); see Fig. 1. A Wilcoxon signed-ranks test indicated that these seasonal differences were statistically significant, *z* = 2.21, *p* = 0.013, *R* = 0.156.

**Fig. 2:**
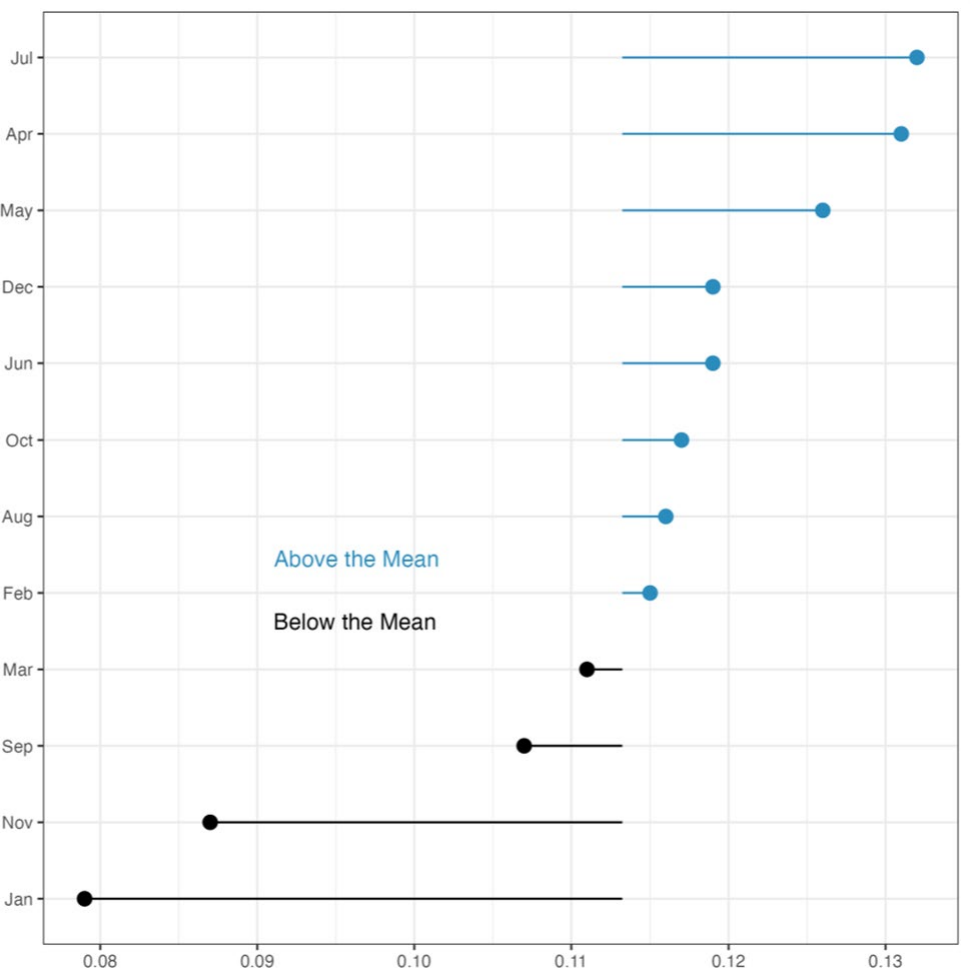
*Monthly Rate of BID per 1000 Tweets for the Northern Hemisphere Panel.* Summer months are Jun, Jul and Aug, with Dec, Jan, and Feb considered winter months

Taking a monthly analysis across all 4 years, July had the highest rate of BID tweets per thousand, providing further support for hypothesis one. August and June also recorded above-average BID rates. Figure 2 details the monthly rate of BID tweets per thousand for the northern hemisphere panel.

### Southern hemisphere

**Fig. 3:**
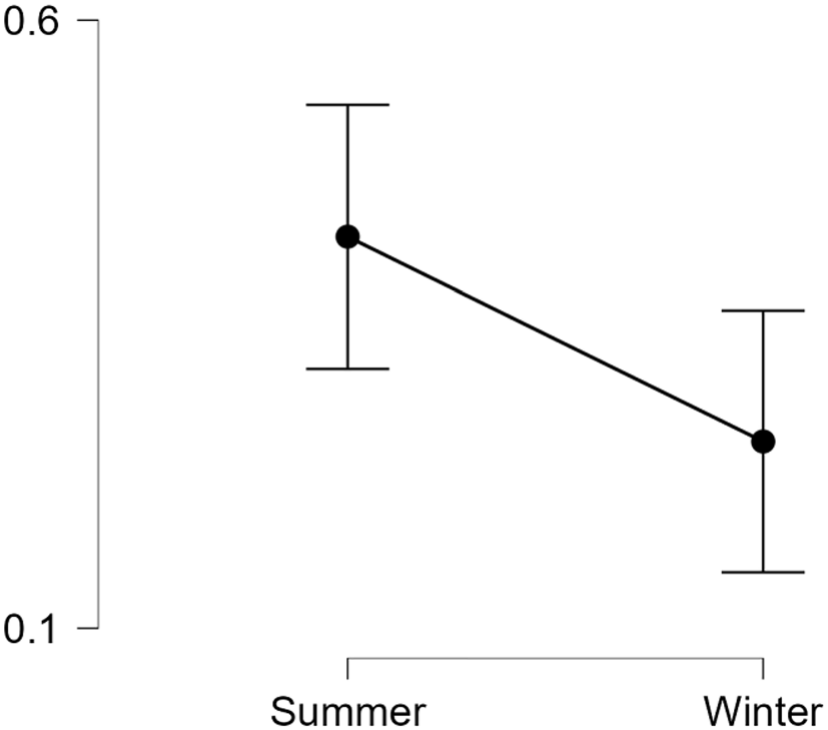
*Mean BID Tweets per 1000 by Season for the Southern Hemisphere Panel.* Summer months are Dec, Jan, and Feb, with Jun, Jul, and Aug considered winter months

In the Southern Hemisphere, the rate of BID tweets was higher during the summer (*MDN* = 0.00303) compared to winter (*MDN* = 0.00295); see Fig. 3. This pattern is directionally supportive of the hypothesis. However, a Wilcoxon signed-ranks test indicated that, for the southern hemisphere, these seasonal differences were not statistically significant.

**Fig. 4:**
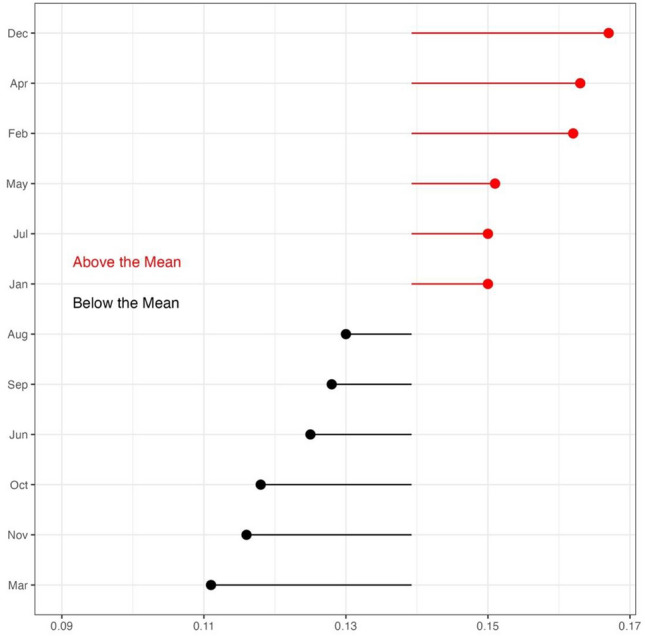
*Monthly Rate of BID per 1000 Tweets for the Southern Hemisphere Panel.* Summer months are Dec, Jan, and Feb, with Jun, Jul, and Aug considered winter months

Beyond the seasonal analysis (summer vs. winter), the monthly examination of the Southern Hemisphere offers partial support for Hypothesis Two, as the highest rate of BID tweets in the region was recorded in December, with both January and February also reporting above-average mean rates. Figure 4 illustrates the monthly rate of BID tweets per thousand for the Southern Hemisphere panel.

### Sex differences

Pooling all data, sex differences were observed in the rates of BID tweets per 1000, with women (*N* = 767, *M* = 0.12, *SD* = 0.45) expressing higher rates than men (*N* = 723, *M* = 0.07, SD = 0.28). A Mann–Whitney test was also employed to further explore assumed sex differences in BID tweet rates. BID rates were significantly higher for women than for men (*U* = 297,840, *p* = 0.06, *R* = 0.074). The median rate for women was 0.0000238, compared to a median of 0.0000117 for men.

## Discussion

This study examined the seasonal body image hypothesis, specifically whether body image dissatisfaction intensifies during the summer, regardless of whether one resides in the Northern or Southern Hemisphere. In line with an earlier cross-sectional exploration (Griffiths et al., 2021), the findings from both hemispheres supported the present study’s hypotheses. However, the summer spike for the Southern Hemisphere did not reach statistical significance.

Equally supportive of seasonal body image hypotheses, a month-by-month analysis revealed that BID tweets were most frequent during the summer months of July and December in the northern and southern hemispheres, respectively. Taken together, these findings further support the notion of seasonal within-person variation in body image, particularly the idea that body image dissatisfaction (BID) is heightened during the summer months. Previous research using Twitter data by Griffiths et al. has also noted an intensification of diet-related social media content during the spring [46]. Griffiths et al. contend that this increase in diet-related content during the spring is a response to prior experiences of heightened summertime body dissatisfaction—a prospective attempt to avoid the anticipated BID associated with the upcoming summer season.

There are likely to be diverse and inter-related drivers of the observed summer spike in BID. One such driver may include intensified pressures—implicit and explicit—to conform to demanding body image standards during this time of year. In line with Griffiths et al. [33], we propose that these pressures may be exerted synergistically by the media, peers, and seasonal social norms. For instance, there may be social expectations to visit the beach during the summer and to wear more revealing clothing; body-confident peers might post more body-focused content on social media, while advertisers may utilise similar imagery to sell seasonal products, and social media platforms often promote seasonally themed body image ideals through hashtags, such as #SummerGoals and #BeachBodyReady. All of this potentially creates the “perfect storm” for upward social comparisons related to weight and shape (comparing one’s body shape/size with images on social media portraying individuals perceived as having superior physiques), resulting in body image dissatisfaction [32].

Focusing specifically on social media, Ryding & Kuss [47] found evidence that both passive (i.e., consuming content without interacting with others) and appearance-based (i.e., exposure to idealised appearance content) social media engagement drove body image dissatisfaction in users. This relationship may be indirectly mediated by upward social comparison [48, 49]. Moreover, there is evidence that receiving online social reinforcement (e.g., likes, comments, or reposts) of curated, photo-edited (airbrushed) “selfies” or “fitspiration”-style content may increase body dissatisfaction in a dose-responsive manner [50, 51].

Beyond the hypothesised summer spikes noted in the present study, April also saw an intensification of BID. It accounted for the second-highest rates of BID tweets in both the northern and southern hemisphere panels, reflecting spring and autumn, respectively. This suggests that, in addition to climatic seasonal variations, traditional holiday periods, such as Easter (spring break), are associated with increased online expressions of BID. In line with this idea, it is important to note that December (winter break/Christmas holidays) in the Northern Hemisphere also experienced a relatively high rate of BID (fourth-highest incidence). This relative increase in online expressions of BID during the holiday season may be attributed to unwanted weight gain [52], heightened expectations surrounding social gatherings, and the associated pressure to pose for photographs [53]. In addition, Park et al. [54] discovered that diet-related online search volume peaked in January in both hemispheres, potentially linked to dissatisfaction with weight gain following the holidays and the setting of New Year’s resolutions and fitness goals for the coming year [55]. Aside from climatic seasonal pressures, the present study supports the notion that BID is also connected to traditional holiday periods.

One limitation of previous research exploring the seasonal body image hypothesis is that it was confined to men [33], whereas the present study also includes women. Research utilising traditional self-report measures typically finds higher levels of BID among women [14], which was also evident in the current analysis. However, it is worth noting that sex/gender is not explicitly recorded on Twitter and has, therefore, been deduced from user biographies where possible.

Another strength of the current study is the use of panel data spanning 4 years, suggesting that the seasonal patterning is enduring (not specific to a particular year). Furthermore, using social media posts instead of self-report data adds a dimension of ecological validity, safeguarding the study against criticisms concerning researcher demand characteristics and socially desirable responses [56].

Overall, the present findings generally support previous research on body image, providing additional evidence for the nascent concept of seasonal body image dissatisfaction [33]. The study also supports the utility of social media as a viable tool for psychological and public health research [39]. This study, of course, has several limitations. One obvious limitation was the discrepancy in panel sizes, particularly the Southern panel’s failure to reach the intended quota. The north–south panel size discrepancy can be attributed to the varying availability of location data for Twitter users, which is dependent on geography. In addition, and more importantly, *X* usage is lower in Australia, New Zealand, and South Africa compared to the United States and the United Kingdom.

Another study limitation, inherent in using social media data, is the possibility that individuals do not always express their authentic feelings; in some cases, they may be joking (being ironic), expressing sarcasm, or even quoting song lyrics (resulting in false positives). This imperfect heuristic-based sentiment assignment (for example, categorising tweets as expressing BID) is practicable, because the typically large sample sizes in social media-based studies safeguard genuine effects from being easily obscured by minor extraneous factors such as noisy data [36, 57, 58].

Although the sample size in this study was relatively large, it was constrained by data access limitations and the availability of personnel for data verification (tagging). The initial tagging (removing business and professional accounts discussing body image) was carried out by a single individual, which prevented the establishment of inter-rater reliability. Future investigations might utilise multiple taggers along with larger data sets. Greater data access would enable researchers to integrate content from multiple social media platforms over longer intervals. This may also include individuals from diverse climates (e.g., equatorial and tropical) and various cultural and language groups (e.g., Arabic and Mandarin). However, based on the current findings, along with those of Griffiths et al. [33], we suggest that interventions targeting body dissatisfaction on English-speaking social media may be particularly warranted during the summer months, when BID expressions peak. Similarly, timing interventions with holiday periods (Christmas and Easter) may also prove beneficial for the same reason. Utilising social media platforms, where BID is expressed, to deliver body positivity campaigns may also be effective. Previous work has shown that disseminating health promotion campaigns through Twitter can be a practical avenue for attitudinal change [59, 60]. The present study offers further tentative support for the seasonal variation hypothesis with potential implications for public health policy aimed at reducing eating disorders and body dysphoria, particularly in the online Anglosphere.

## Supplementary Information

Below is the link to the electronic supplementary material.

Supplementary file 1.

## Data Availability

The anonymised data sets analysed during the current study are available from the corresponding author on request.

## Declarations

### Strength and limits

This study employed a relatively large sample and can boast ecological validity, as subjects used social media in a naturalistic setting, free from demand characteristics. The 4-year span of the study is also a strength. Limitations include sample size issues for the southern hemisphere. The study is also correlational, so it can tell us nothing definitive about causation or possible mechanisms that explain the seasonal trends.

### What is already known on this subject?

Based on one earlier study, a self-report survey with a limited sample, we know that body image dissatisfaction among sexual-minority males appears to follow a seasonal pattern. This was based on a 1-year follow-up. This study extends the concept of seasonal body image to a more diverse sample and confirms the pattern across multiple years.

### What this study adds?

We now have greater certainty that body image dissatisfaction follows a seasonal pattern, with hotter months associated with greater levels of body dysphoria. However, there also appear to be spikes associated with traditional holiday periods, such as Easter (spring break) and Christmas.
