# Supplementary figures and images for: Seasonal body image dissatisfaction: a bi-hemispheric panel analysis of social media users across 4 years
Source: Eat Weight Disord. 2025 Aug 31;30(1):72. doi: 10.1007/s40519-025-01782-9 (PMC12399709; doi:10.1007/s40519-025-01782-9)

Appendix 1: Search String Generated


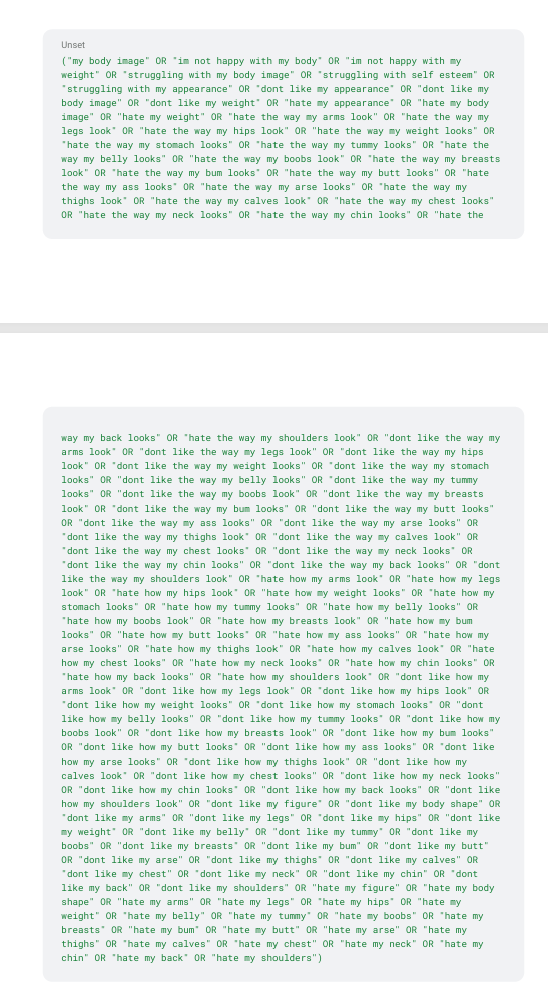

Supplement: Supplementary file 1 [file 40519_2025_1782_MOESM1_ESM.docx]
